# What We Know and What We Don't Know About Preventing Stroke

**DOI:** 10.1371/journal.pmed.1001637

**Published:** 2014-04-29

**Authors:** Druin Burch, the PLOS Medicine Editors

## Abstract

Consulting Editor Druin Burch and the *PLOS Medicine* Editors discuss implications of research by Bos and colleagues, which finds that a high proportion of strokes in a European cohort are not attributable to known risk factors.

*Please see later in the article for the Editors' Summary*

“Stroke” was a word used in a variety of medical senses from ancient times, but it acquired its modern medical meaning by the late sixteenth century: the word implied a cerebrovascular accident, a sudden event. Modern primary and secondary prevention has reframed stroke as the abrupt end result of chronic processes that can be understood and retarded. Despite improvements in treatment and prevention, and despite a decline in age-standardized mortality, the global burden of stroke continues to rise [1]. Older and larger populations, less subject to infectious diseases, mean more people than ever before are dying from strokes or living with consequent disability. In research published this week in *PLOS Medicine*, Bos and colleagues [2] highlight remaining uncertainty over some of the causes of cerebrovascular disease.

The Framingham Heart Study, which began recruitment in 1948 [3], altered the perception of cardiovascular disease as being an unavoidable cause of early death and disability, identifying the importance of blood pressure, cholesterol, smoking, diabetes, weight, and inactivity. Framingham gave rise to our familiarity with the term “risk factors” [4], established the importance of atrial fibrillation for stroke, and suggested the potential benefits of anticoagulants or rhythm control [5].

Bos and colleagues make no attempt to bring new risk factors to light but their study suggests there may be a need to do so. Their paper finds that the main known risk factors, taken together, explain a smaller proportion of strokes than previous studies had indicated. INTERSTROKE [6], a case-control study investigating 22 high-, middle-, and low-income countries, found known risk factors explained 90% of all strokes, a figure roughly consistent with other investigations [7]. Bos and colleagues attribute only 51% of strokes to these main known causes. Differences in methodology may contribute to this variance, but neither the present study's statistical sophistication nor its cohort design seem likely to wholly explain why these population attributable risks (PARs) are so much lower than previous estimates.

One intriguing possibility is the peculiar nature of the Rotterdam population, which was far more homogeneous than those of INTERSTROKE. Low- and middle-income countries (LMICs) bear two-thirds of the global burden of stroke and stroke-related deaths [1], but those studied in Rotterdam were relatively wealthy, even in the context of the Netherlands. Access to health care was financially unrestricted and enrollment in the study raised awareness of existing risk factors. Participants were substantially older (a median of 68 years of age on recruitment, followed by a mean 13 years of follow-up) and, as the authors point out, “the relation between stroke and its etiological factors weakens with increasing age.” Taken together, these issues suggest that while this study may currently be of limited generalizability, it could also be a window on the future. Improvements in access to health care, and an increase in wealth, stand to make Rotterdam's experience more common.

PARs are dependent on the prevalence of risk factors and of the relative risk associated with each. In the Rotterdam population studied by Bos and others, hypertension prevalence was comparable to INTERSTROKE but diabetes was significantly lower, present in only 10% of the cohort rather than 24% of INTERSTROKE controls. There was almost a 4-fold increase in stroke risk in INTERSTROKE for those with a blood pressure over 160/90 mm Hg, a figure consistent with prospective studies [8], but in Rotterdam, the risk associated with hypertension was roughly half that. Diabetic risk too seems ameliorated in Rotterdam, and while the relative risk of 1.43 is similar to the 1.36 of INTERSTROKE, both appear conspicuously lower than 1.83 in men and 2.28 in women suggested by a recent meta-analysis of cohort studies [9]. Bos and colleagues found no association between stroke and body mass index, but the lack of measurement of waist-hip-ratio may have been an important omission: INTERSTROKE found waist-hip-ratio to account for a PAR of 26% and that it was more strongly associated with stroke risk than body mass index. Some differences between INTERSTROKE and the Rotterdam study are likely to be due to an older population in the latter, but improved treatment of risk factors as the result of above average wealth, health care, and awareness may matter too.

## What Needs to Be Done to Prevent Stroke?

Also published this month in *PLOS Medicine*, a study of 21 cohorts comprising more than 1 million participants in several Asian countries found the prevalence of tobacco smoking to be 65.1% among male and 7.1% among female participants [10]. Across LMICs in general, 48% of men use tobacco [11].

Assuming the relative risk associated with current smoking found by INTERSTROKE is generalizable, the PAR for stroke in LMICs would be 36%—meaning that smoking could cause a third of all strokes in those countries. Tobacco control remains the chief global priority, followed next by hypertension. In the US only 69% of adults with hypertension are aware of it and only half of those on treatment are controlled [12]. In LMICs hypertension prevalence is similar but awareness and effective treatment rates are very much lower [13].

Action to control known risk factors, particularly tobacco and hypertension, remains the chief priority for stroke prevention. Physical activity, another modifiable risk factor, was not assessed in the study by Bos and colleagues, but it is also an important target The decline in PARs from existing risk factors, as populations become older, better treated, healthier, and more like that of Rotterdam, will raise the relative importance of other causal pathways. For example, although neither total nor non-high-density lipoprotein cholesterol are associated with increased risk of stroke in observational studies [9], evidence from randomized controlled trials suggests that statins may confer a degree of protection against stroke independent of baseline low-density lipoprotein level [14]. More generally, large exploratory studies with broad strategies for collecting phenotypic, genetic, and outcome data are underway. Biobank in the UK and the China Kadoorie Biobank (particularly with regards to that country's higher rate of hemorrhagic stroke) may represent the modern equivalent of the work started in 1948 in Framingham. As the chief known risk factors for stroke come under control, others will necessarily become more important. Future stroke prevention strategies will need to be based on understanding ever-shifting patterns of PAR.

## Abbreviations

LMIC: low- to middle-income country
PAR: population attributable risk
